# *Escherichia coli* resistance, treatment patterns and clinical outcomes among females with uUTI in Germany: a retrospective physician-based chart review study

**DOI:** 10.1038/s41598-023-38919-8

**Published:** 2023-07-26

**Authors:** Kurt G. Naber, Florian Wagenlehner, Michael Kresken, Wendy Y. Cheng, Maryaline Catillon, Mei Sheng Duh, Louise Yu, Anamika Khanal, Aruni Mulgirigama, Ashish V. Joshi, Shinyoung Ju, Fanny S. Mitrani-Gold

**Affiliations:** 1 Technical University of Munich, Munich, Germany; 2 Clinic of Urology, Pediatric Urology and Andrology, Justus Liebig University Giessen, Giessen, Germany; 3 Antiinfectives Intelligence GmbH, Cologne, Germany; 4 Analysis Group, Inc., Boston, MA USA; 5 GSK, Brentford, Middlesex UK; 6 GSK, 1250 S Collegeville Road, Collegeville, PA 19426 USA

**Keywords:** Antimicrobial resistance, Urinary tract infection

## Abstract

Real-world data were collected to examine antimicrobial resistance (AMR) prevalence, treatment patterns, and clinical outcomes among female patients with uncomplicated urinary tract infection (uUTI) in Germany. Data were from a retrospective physician-based chart review completed by physicians treating patients with uUTI. Non-pregnant women aged ≥ 12 years, with a uUTI diagnosis, an *E. coli-*positive urine culture between January 2017–December 2019, and susceptibility test results for ≥ 4 drug classes were eligible. Patients were stratified into three cohorts by drug class susceptibility: susceptible to all (SUS), resistant to one or two drug classes (DR1/2), and resistant to ≥ 3 (MDR) drug classes tested. Among 386 eligible patients [SUS (67.1%); DR1/2 (29.0%); MDR (3.9%)], AMR prevalence was highest for FMIs (18.3%) and lowest for fluoroquinolones (5.2%). The most prescribed drugs were fosfomycin in SUS (44.0%), DR1/2 (41.4%), and fluoroquinolones in MDR (40.0%). Treatment for uUTI failed for 8.8% of patients; failure was more likely in MDR versus SUS [adjusted odds ratio [95% CI] = 4.21 [1.14–1.50]; P = 0.031); incidence of recurrent infection in the 6-months post-index period was higher in DR1/2 versus SUS. These findings may have implications for empiric prescribing, suggesting an unmet need for new treatments.

## Introduction

Urinary tract infections (UTIs) are more common in women than men^1^, and recurrent infections can be associated with significant physical and psychological burden^2^. Uncomplicated UTIs (uUTIs; also known as acute uncomplicated cystitis [AUC]), often caused by *Escherichia coli* (*E. coli*)^3,4^, are among the most frequently encountered infections among females in the outpatient setting^5,6^. Current UTI treatment guidelines recommend empiric antimicrobial prescribing for uUTIs, but frequent and inappropriate antimicrobial use increases the risk of antimicrobial resistance (AMR)^3,4,7,8^.

Varying trends of AMR among *E. coli* isolates have been observed in recent decades^9^, and some data indicate that the prevalence of AMR is on the rise. For example, previous estimates of prevalence of resistance to ciprofloxacin in Germany suggest resistance has increased from 8.5% in 2011^10^ to 15.1% between 2015 and 2017^11^. Worldwide, prevalence of AMR has been associated with an estimated 4.95 million deaths in 2019, including 1.27 million deaths directly attributable to resistance^12^. AMR trends in Germany are projected to increase further, as in many other countries, if no effective action is put in place^13^. However, up-to-date evidence on single and multiple drug resistance in Germany is scarce, and real-world data on treatment patterns, healthcare resource utilization, healthcare costs, and clinical outcomes are limited^3^. Specific knowledge of regional and local resistance is required for optimizing empiric uUTI prescribing and treatment.

The study objectives were to assess the prevalence of AMR, patient characteristics (demographic and clinical), treatment patterns, and clinical outcomes among females with uUTI caused by *E. coli* in Germany.

## Methods

### Study design

**Figure 1:**
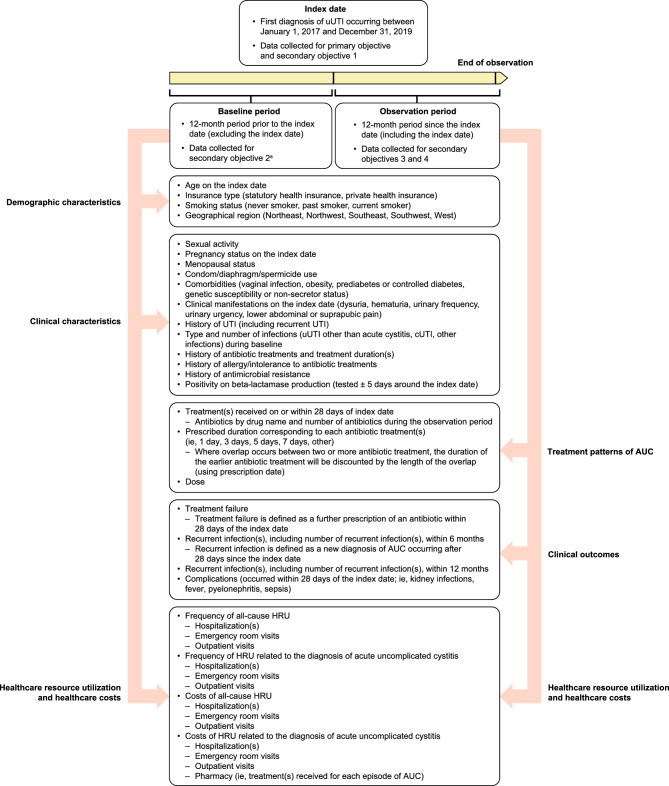
Study design. ^a^Some baseline characteristics (such as age and pregnancy status) collected on index date only. *AUC* acute uncomplicated cystitis, *HRU* healthcare resource utilization*, cUTI* complicated urinary tract infection, *UTI* urinary tract infection, *uUTI* uncomplicated urinary tract infection.

This retrospective, physician-based chart review study used data abstracted by German physicians who treated uUTI (Fig. 1). The index date was defined as the first documented uUTI diagnosis based on positive urine culture with *E. coli* occurring between January 1, 2017, and December 31, 2019, with a baseline period 12-months prior to the index date and an observation period 12-months after the index date. The 12-month baseline period was required to be able to assess history of prior resistance, prior antimicrobial treatment, comorbidities, and to apply exclusions for complicated UTI. The prevalence of AMR (i.e., resistance to one, two, and three or more drug classes) was based on urine culture and antimicrobial susceptibility test results, which were collected within 5 days of the index date to link the UTI diagnosis to the culture and susceptibility. Susceptibility testing for the isolate was performed by each laboratory according to the European Committee on Antimicrobial Susceptibility Testing (EUCAST) criteria using either standard commercial panels for urine cultures or via disc diffusion methods.

The primary objective was to assess the prevalence of AMR among female outpatients with uUTI caused by *E. coli*, with stratification by antimicrobial susceptibility into three cohorts: susceptible to all drug classes tested (SUS); resistant to one or two drug classes tested (DR1/2); and resistant to ≥ 3 drug classes tested (MDR). Secondary objectives were: (1) to assess the prevalence of AMR by geographic region; (2) to assess and compare demographics and clinical characteristics of patients with uUTI across the three antimicrobial susceptibility cohorts; (3) to assess and compare treatment patterns across three antimicrobial susceptibility cohorts; and (4) to assess and compare clinical outcomes of patients with uUTI across three antimicrobial susceptibility cohorts. The antimicrobial drug classes recommended as first choice by the German UTI treatment guidelines^15^ are: (1) fosfomycin-trometamol (active drug fosfomycin); (2) nitrofurantoin; (3) pivmecillinam (active drug mecillinam); (4) nitroxoline; and (5) trimethoprim (TMP; if local resistance is < 20%). The German guidelines do not recommend TMP/sulfamethoxazole (SXT), cefpodoxime-proxetil or fluoroquinolones as first choice antibiotics. Patients' treatment patterns were assessed within 28 days of the index date and included both the first antibiotic prescribed (empiric therapy) and culture-guided therapy (when applicable). Antibiotic treatments received on the same day (concurrent antibiotic therapy) were differentiated from treatments received on different days within the 28-day follow-up period. Patients with single episode of treatment were also differentiated from those with multiple episodes of treatment.

The study protocol was approved by a central Institutional Review Board (exempt according to FDA 21 CFR 56.104 and 45CFR46.104[b][4]: [4] Secondary Research Uses of Data or Specimens on 05/21/2021) prior to commencement of data collection and was registered with German authorities under BfArM internal NIS number 7514, according to section 67 subsection 6 of the German Medicinal Products Act. The study was conducted entirely using retrospective medical records. No patient identifiable information was collected, and data were fully anonymized in compliance with General Data Protection Regulation (GDPR) in the European Union; because the data collected were anonymized according to Recital 26 of GDPR, the regulation’s patient informed consent requirements were not applicable (see European Union Regulation 2016/679, Recital 26). Anonymized data collected from physicians were transferred to study project personnel via a secure, encrypted network and were stored in secure servers. Minimum retention time complied with all applicable laws and regulations. Participating physicians were compensated per local fair market value rates for the time it took to abstract the patient chart.

### Study population

Medefield, a physician panel vendor with access to a large panel of registered physicians, was contracted to recruit physicians to conduct a physician-based medical chart review in Germany. Physicians from three medical specialties were recruited: urology, general medicine/internal medicine/primary care, and obstetrics/gynecology. Participating physicians had to have access to complete medical records for ≥ 1 patient who met the patient eligibility criteria and each physician abstracted data for 1–3 patients. In the case that > 3 patients were available to a single physician, the three included patients were chosen randomly using a random letter generator in the electronic case report form. The inclusion criteria for eligible patients were: female outpatients, ≥ 12 years of age at index with a confirmed diagnosis of community-acquired uUTI (AUC) based on a positive urine culture with *E. coli* between January 1, 2017, and December 31, 2019 (the date of the first diagnosis of uUTI during this period is the index date), a urine susceptibility test performed with results available within 5 days of the index date, and ≥ 12-months of medical history prior to (baseline period) and after (observation period) the index date. Patients were excluded if they had: asymptomatic bacteriuria; symptomatic uUTI with complicating comorbidities (uncontrolled or complicated diabetes mellitus [hemoglobin A1c > 7]; immunosuppression or were treated with immunosuppressants; relevant urological abnormalities during baseline/at the index date); received relevant urological or nephrological procedures (e.g., catheter, stent, or surgery) during the baseline period; or received intravenous antimicrobial treatment/were hospitalized on or within 4 weeks prior to the index date.

### Study outcomes

The following clinical outcomes were collected during the 12-month observation period and compared between cohorts: treatment failure (defined as an additional prescription of an antimicrobial for acute cystitis within 28 days of the index date), recurrent infections (including the number of infections occurring within 6 and 12 months of index), and UTI complications (occurring within 28 days of the index date such as fever, pyelonephritis, and sepsis). Differences in baseline characteristics and treatment patterns across cohorts were described using standardized differences (std. diffs). Univariable and multivariable logistic regressions and Poisson regressions were used to compare clinical outcomes separately for DR1/2 versus SUS cohorts and MDR versus SUS cohorts.

For binary outcomes, adjusted odds ratios (ORs) were estimated, and for count outcomes adjusted incidence rate ratios (IRRs) were reported. The multivariable model was adjusted for age at index, prior history of UTI status (pre-index), and receipt of antimicrobial treatment during the 12-month baseline period. Ninety-five percent confidence intervals (95% CIs) were reported for all estimates. All analyses were conducted using SAS, Version 9.4 (SAS Institute, Cary, NC).

### Variables and statistical analyses

The following variables were collected from physicians participating in the study: medical specialty (urology, general medicine/internal medicine/primary care, obstetrics/gynecology); geographical region (Northeast, Northwest, Southeast, Southwest, West); and practice setting (office-based, outpatient hospital-based, mixed). The following patient characteristics were collected, irrespective of antimicrobial susceptibility test results: baseline demographics (age, insurance type, smoking status, and geographical region) and clinical characteristics [sexual activity, pregnancy, menopausal status, vaginal infection, obesity, pre- or controlled diabetes mellitus (hemoglobin A1c ≥ 5.7 and ≤ 7), prior UTI, history of recurrent UTI (≥ 2 uUTIs in 6-months or ≥ 3 in 1 year)]. Other patient characteristics collected were hospitalization, emergency department visits, antimicrobial treatment (in the 12-month period prior to index date), type and number of infection episodes during baseline, allergy or intolerance by antimicrobial drug class, history of antimicrobial treatments, and AMR. Information on clinical manifestations on the index date (i.e., dysuria, urinary frequency/urgency, lower abdominal or suprapubic pain), and positive beta-lactamase production on or around the index date were collected. Antimicrobial susceptibility results on or around the index date, and treatment patterns (treatments received on or within 28 days post index date, and drug dose and duration) were also collected.

A formal power calculation was not performed because this was a descriptive study, with no hypothesis being tested. We performed an analysis to estimate the precision around 95% CIs for the primary objective or prevalence of AMR. With a sample size of n = 400, the 95% CI ranged from 45 to 55% (width of 10%) at the highest AMR proportion of 50%. This precision estimate was based on expected AMR prevalence in Germany ranging from 2 to 50% (fosfomycin-trometamol: 2%, nitrofurantoin: 5%, cefpodoxime-proxetil: 7.1%, TMP-SMX: 25–36.8%, ciprofloxacin: 8.2%) based on surveillance data and expanded to a hypothetical 50% (13.2% higher than any AMR rate reported in German surveillance^11,16–19^). Though data were gathered to address secondary objectives, the small size of the MDR cohort limited us in detecting meaningful differences between cohorts regarding treatment patterns and clinical outcomes. Pregnant patients were included descriptively in analyses of AMR by region (with stratification), but were not included in the subgroup or cohort analyses.

### Compliance with ethical standards

The study protocol was approved by a central Institutional Review Board prior to commencement of data collection (#21-ANGR-102, Pearl IRB, IN, USA). The study was conducted entirely using retrospective medical records. No patient identifiable information was collected, and data were fully anonymized in compliance with General Data Protection Regulation in the European Union; thus, informed consent was not required according to FDA 21 CFR 56.104 and DHHS 45 CFR 46.104(b)(4): Secondary Research Uses of Data or Specimens (Pearl IRB, IN, USA). Anonymized data collected from physicians were transferred to project personnel via a secure, encrypted network and were stored in secure servers. Minimum retention time complied with all applicable laws and regulations.

## Results

### Physician characteristics

**Figure 2:**
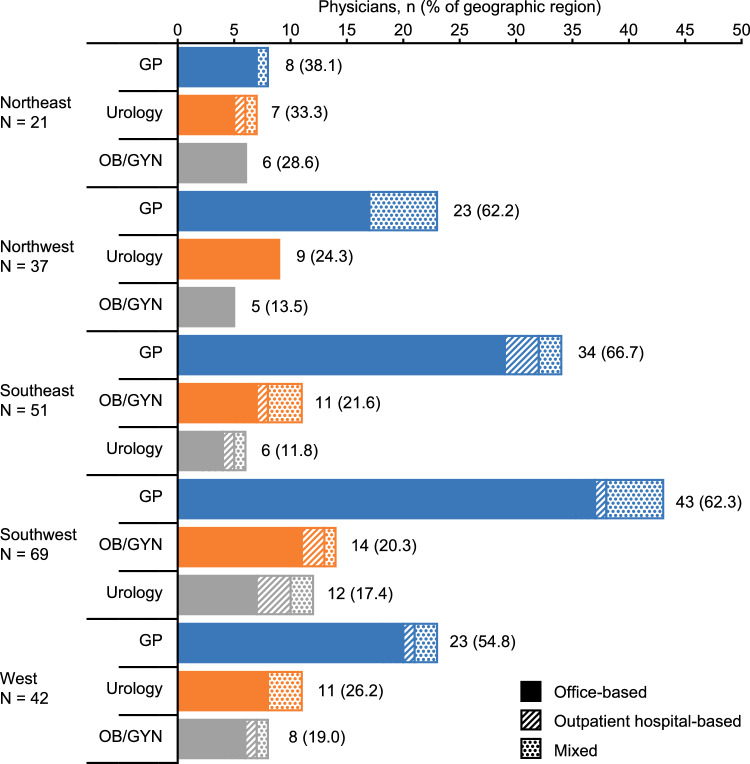
Regional distribution of physicians by region and work setting. Each physician completed an electronic case report form for at least one and up to three patients. The following Federal States were included in each region: Northeast (Berlin, Brandenburg, Mecklenburg-Western Pomerania, and Saxony-Anhalt), Northwest (Schleswig-Holstein, Hamburg, Lower Saxony, and Bremen), Southeast (Bavaria, Thuringia, and Saxony), Southwest (Hesse, Rhineland-Palatinate, Baden-Württemberg, and Saarland), and West (North Rhine-Westphalia). *GP* general practitioner, *OB* obstetrician, *GYN* gynecologist.

A total of 220 treating physicians abstracted patient charts retrospectively; physician characteristics are shown in Fig. 2. Most physicians were general practitioners (n = 131; 59.5%); 45 (20.5%) were urologists and 44 (20.0%) specialized in obstetrics/gynecology. The majority of physicians were office-based (n = 178; 80.9%). The study included physicians from all five German geographic regions (Fig. 2). On average, physicians completed two charts each.

### Patients

In total, 438 patients met the inclusion criteria. Of these, 46 patients had *E. coli* isolates that had not undergone antimicrobial susceptibility testing for ≥ 4 drug classes and six patients were pregnant at the index date. Pregnant patients were excluded from the analyses to align with the European Association of Urology guidelines, which state that uUTIs are acute, sporadic, or recurrent lower UTIs, which are limited to non-pregnant women with no known relevant anatomical and functional abnormalities within the urinary tract or complicating comorbidities^4^. A total of 386 non-pregnant patients were eligible and included in the remaining analyses.

### Demographic and clinical characteristics by susceptibility cohort

Baseline patient demographics and clinical characteristics are shown in Table 1. Of 386 non-pregnant female patients, the overall mean [standard deviation (SD)] age was 41.7 (16.7) years: the MDR cohort of patients were older [47.3 (16.8) years] than the SUS cohort [43.0 (17.0) years] and the DR1/2 cohort [37.9 (15.2) years]. A higher proportion of patients in the MDR cohort had a prior history of UTIs (n = 6; 40.0%) compared with the SUS (n = 58; 22.4%) and DR1/2 (n = 26; 23.2%) cohorts. Overall, most patients were premenopausal (n = 273; 70.7%) and sexually active (n = 281; 72.8%) at the time of uUTI diagnosis. A greater proportion of the DR1/2 cohort had obesity (n = 26; 23.2%) and prior vaginal infections during baseline (n = 22; 19.6%), compared with the SUS cohort, in which 38 (14.7%) patients had obesity and 30 (11.6%) patients had prior vaginal infections. A larger proportion of patients in the MDR cohort experienced dysuria (n = 15; 100%) and hematuria (n = 10; 66.7%), compared with the SUS cohort: dysuria (n = 230; 88.8%) and hematuria (n = 134; 51.7%). A higher percentage of MDR patients (n = 14; 93.3%) had no prior antimicrobial intolerance, compared with the SUS (n = 229; 88.4%) and DR1/2 (n = 97; 86.6%) cohorts. Other patient characteristics, including history of antimicrobial treatments, history of antimicrobial allergy/intolerances, and history of AMR, are reported in Supplementary Table 1**.** The frequency and proportion of patients with at least one hospitalization for both the baseline and observation periods were measured across all cohorts. During the baseline period, seven patients (9.0%) had at least one all-cause hospitalization, with a mean length of stay of 7 days; no patient was hospitalized due to a UTI complication (acute pyelonephritis or sepsis).

**Table 1:** Baseline demographics and clinical characteristics among non-pregnant female patients with uUTI and AMR information for ≥ 4 drug classes.

Baseline demographics and clinical characteristics	All patients (N = 386)	Antimicrobial susceptibility cohort			Std. diff^a,b^, %	
		[1] SUS (n = 259)	[2] DR1/2^c^ (n = 112)	[3] MDR^c^ (n = 15)	[2] vs [1]	[3] vs [1]
Mean (SD) age^d^, years	41.7 (16.7)	43.0 (17.0)	37.9 (15.2)	47.3 (16.8)	31.9^e^	25.1^e^
Region, n (%)						
Northeast	38 (9.8)	23 (8.9)	13 (11.6)	2 (13.3)	9.0	14.2
Northwest	57 (14.8)	36 (13.9)	20 (17.9)	1 (6.7)	10.8	24.0^e^
Southeast	90 (23.3)	63 (24.3)	24 (21.4)	3 (20.0)	6.9	10.4
Southwest	119 (30.8)	81 (31.3)	32 (28.6)	6 (40.0)	5.9	18.3
West	82 (21.2)	56 (21.6)	23 (20.5)	3 (20.0)	2.7	4.0
Statutory health insurance^f^, n (%)	320 (82.9)	213 (82.2)	94 (83.9)	13 (86.7)	4.5	12.2
Smoking status, n (%)						
Never smoked	164 (42.5)	114 (44.0)	42 (37.5)	8 (53.3)	13.3	18.7
Past smoker	99 (25.6)	66 (25.5)	30 (26.8)	3 (20.0)	3.0	13.1
Current smoker	76 (19.7)	46 (17.8)	27 (24.1)	3 (20.0)	15.7	5.7
Unknown	47 (12.2)	33 (12.7)	13 (11.6)	1 (6.7)	3.5	20.6^e^
Premenopausal^d^, n (%)	273 (70.7)	179 (69.1)	86 (76.8)	8 (53.3)	17.3	32.8^e^
Sexually active^d^, n (%)	281 (72.8)	177 (68.3)	94 (83.9)	10 (66.7)	37.2^e^	3.6
Comorbidities^d^, n (%)						
Vaginal infections	53 (13.7)	30 (11.6)	22 (19.6)	1 (6.7)	22.3^e^	17.1
Obesity	68 (17.6)	38 (14.7)	26 (23.2)	4 (26.7)	21.9^e^	30.0^e^
Pre-diabetes or controlled diabetes mellitus^g^	15 (3.9)	11 (4.2)	4 (3.6)	0 (0.0)	3.5	29.8^e^
Clinical manifestations, n (%)						
Dysuria	346 (89.6)	230 (88.8)	101 (90.2)	15 (100)	4.5	50.2^h^
Hematuria	193 (50.0)	134 (51.7)	49 (43.8)	10 (66.7)	16.0	30.7^e^
Urinary frequency	322 (83.4)	216 (83.4)	93 (83.0)	13 (86.7)	1.0	9.2
Urinary urgency	261 (67.6)	172 (66.4)	79 (70.5)	10 (66.7)	8.9	0.6
Lower abdominal or suprapubic pain	170 (44.0)	113 (43.6)	52 (46.4)	5 (33.3)	5.6	21.3^e^
Prior history of infections						
Any prior UTI during baseline period, n (%)	90 (23.3)	58 (22.4)	26 (23.2)	6 (40.0)	2.0	38.7^e^
Median (IQR) number of episodes	2.0 (1.0, 2.0)	2.0 (1.0, 2.0)	2.0 (1.0, 3.0)	2.0 (2.0, 3.0)	-	-
No antimicrobial treatment during baseline, n (%)	265 (68.7)	178 (68.7)	77 (68.8)	10 (66.7)	0.1	4.4
No prior history of antimicrobial intolerance^d,i^, n (%)	340 (88.1)	229 (88.4)	97 (86.6)	14 (93.3)	5.5	17.1
No prior history of AMR^d,j^, n (%)	330 (85.5)	232 (89.6)	87 (77.7)	11 (73.3)	32.6^e^	42.7^e^

Standardized difference > 20%, > 50%, and > 80% denotes a small, medium, and large difference, respectively, between compared groups (per Cohen J. Statistical Power Analysis for the Behavioral Sciences. 2nd ed.; 1988).

*AMR* antimicrobial resistance, *DR1/2* resistant to one or two drug classes, *HbA1c* hemoglobin A1c, *IQR* interquartile range, *MDR* resistant to three or more drug classes tested, *SD* standard deviation, *std. diff* standardized difference*, SUS* susceptible, *TMP* trimethoprim, *UTI* urinary tract infection, *uUTI* uncomplicated urinary tract infection.

^a^For continuous variables, the standardized difference was calculated by dividing the absolute difference in means between cohorts by the pooled standard deviation of both groups (the pooled standard deviation was the square root of the average of the squared standard deviations).

^b^For dichotomous variables, the standardized difference was calculated using the following equation where P was the respective proportion of participants in each group: |(Pcase − Pcontrol)|/√[(Pcase(1 − Pcase) + Pcontrol(1 − Pcontrol))/2].

^c^Uropathogens from patients were considered resistant to a drug class if they were resistant to ≥ 1 drug within the class.

^d^Assessed at index date. ^e^Standardized difference > 20%.

^f^ > 1 health insurance type was permitted—statutory data shown only, the remainder were private health insurance.

^g^HbA1c ≥ 5.7 and HbA1c ≤ 7. ^h^Standardized difference > 50%.

^i^Intolerance to any of the six antimicrobial drug classes tested.

^j^Resistance to agents including fosfomycin, nitrofurantoin, quinolones, beta-lactams, penicillins, macrolides, TMP, and others.

### Prevalence of AMR by drug class (primary objective)

Overall, 386 non-pregnant female patients with uUTI had antimicrobial susceptibility test results for ≥ 4 antimicrobial drug classes; among these, 259 (67.1%) were SUS, 112 (29.0%) were DR1/2, and 15 (3.9%) were MDR (Table 2). The MDR cohort had the highest susceptibility testing (% tested) for most drug classes (80.0–100.0%), compared to SUS (68.7–96.1%) and DR1/2 (69.6–98.2%). Overall, the prevalence of AMR was highest against FMIs (18.3%) and lowest against fluoroquinolones (5.2%). In the DR1/2 cohort, the prevalence of AMR was highest against FMIs (52.6%), and lowest against fluoroquinolones (11.7%) and fosfomycin (10.0%). In the MDR cohort, the highest prevalence of AMR was observed for mecillinam and FMIs (both 66.7%), and lowest against fluoroquinolones and cefpodoxime (both 42.9%) (Table 2)**.**

**Table 2:** Antimicrobial resistance among non-pregnant female patients with uUTI who had susceptibility for ≥ 4 drug classes.

Index drug class	All patients (N = 386)	Antimicrobial susceptibility cohort		
		SUS (n = 259)	DR1/2^a^ (n = 112)	MDR^a^ (n = 15)
Fosfomycin				
Tested	373 (96.6)	248 (95.8)	110 (98.2)	15 (100.0)
Susceptible^b^	353 (94.6)	248 (100)	99 (90.0)	6 (40.0)
Resistant	20 (5.4)	0 (0.0)	11 (10.0)	9 (60.0)
Nitrofurantoin				
Tested	368 (95.3)	249 (96.1)	105 (93.8)	14 (93.3)
Susceptible^b^	344 (93.5)	249 (100)	88 (83.8)	7 (50.0)
Resistant	24 (6.5)	0 (0.0)	17 (16.2)	7 (50.0)
Mecillinam				
Tested	364 (94.3)	242 (93.4)	107 (95.5)	15 (100.0)
Susceptible^b^	319 (87.6)	242 (100)	72 (67.3)	5 (33.3)
Resistant	45 (12.4)	0 (0.0)	35 (32.7)	10 (66.7)
Fluoroquinolones^c^				
Tested	345 (89.4)	228 (88.0)	103 (92.0)	14 (93.3)
Susceptible^b^	327 (94.8)	228 (100)	91 (88.3)	8 (57.1)
Resistant	18 (5.2)	0 (0.0)	12 (11.7)	6 (42.9)
Cefpodoxime				
Tested	347 (89.9)	230 (88.8)	103 (92.0)	14 (93.3)
Susceptible^b^	314 (90.5)	230 (100)	76 (73.8)	8 (57.1)
Resistant	33 (9.5)	0 (0.0)	27 (26.2)	6 (42.9)
FMIs^d^				
Tested	268 (69.4)	178 (68.7)	78 (69.6)	12 (80.0)
Susceptible^b^	219 (81.7)	178 (100)	37 (47.4)	4 (33.3)
Resistant	49 (18.3)	0 (0.0)	41 (52.6)	8 (66.7)

*DR1/2* resistant to one or two drug classes, *FMI* folate metabolism inhibitor, *MDR* resistant to three or more drug classes tested, *SUS* susceptible, *SXT* trimethoprim-sulfamethoxazole, *TMP* trimethoprim, *uUTI* uncomplicated urinary tract infection.

^a^Uropathogens from patients were considered resistant to a drug class if they were resistant to ≥ 1 drug within the class.

^b^Susceptible + intermediate/susceptible at higher dose.

^c^Included ciprofloxacin, levofloxacin, and ofloxacin.

^d^Included TMP and SXT.

Resistance by drug-class combinations among the DR1/2 and MDR cohorts is detailed in Table 3. In the DR1/2 cohort, most *E. coli* isolates (72.3%) were resistant to only one drug class tested, most commonly FMIs (26.8%). The highest prevalence of dual-drug class resistance in DR1/2 was against mecillinam + cefpodoxime (6.3%). In the MDR cohort, most isolates were resistant to three drug classes (93.3%); only one isolate was resistant to four drug classes. The most common drug class resistance combinations observed in the MDR cohort were fosfomycin + nitrofurantoin + mecillinam, and fosfomycin + nitrofurantoin + FMIs (both 20.0%).

**Table 3:** Resistance by drug class combinations in the DR1/2 and MDR cohorts.

Drug class combinations	Number of patients
DR1/2	N = 112 (100)
Resistant to one drug class, n (%)	81 (72.3)
FMIs	30 (26.8)
Mecillinam	19 (17.0)
Cefpodoxime	13 (11.6)
Nitrofurantoin	9 (8.0)
Fosfomycin	6 (5.4)
Fluoroquinolones	4 (3.6)
Resistant to two drug classes, n (%)	31 (27.7)
Mecillinam + cefpodoxime	7 (6.3)
Mecillinam + FMIs	6 (5.4)
Fluoroquinolones + FMIs	4 (3.6)
Fosfomycin + nitrofurantoin	3 (2.7)
Nitrofurantoin + cefpodoxime	3 (2.7)
Fluoroquinolones + cefpodoxime	2 (1.8)
Nitrofurantoin + mecillinam	2 (1.8)
Mecillinam + fluoroquinolones	1 (0.9)
Fosfomycin + fluoroquinolones	1 (0.9)
Fosfomycin + cefpodoxime	1 (0.9)
Cefpodoxime + FMIs	1 (0.9)
MDR	N = 15 (100)
Resistant to three drug classes, n (%)	14 (93.3)
Fosfomycin + nitrofurantoin + mecillinam	3 (20.0)
Fosfomycin + nitrofurantoin + FMIs	3 (20.0)
Mecillinam + fluoroquinolones + FMIs	1 (6.7)
Mecillinam + fluoroquinolones + cefpodoxime	1 (6.7)
Mecillinam + cefpodoxime + FMIs	1 (6.7)
Fluoroquinolones + cefpodoxime + FMIs	1 (6.7)
Fosfomycin + mecillinam + fluoroquinolones	1 (6.7)
Fosfomycin + mecillinam + cefpodoxime	1 (6.7)
Fosfomycin + fluoroquinolones + cefpodoxime	1 (6.7)
Nitrofurantoin + mecillinam + FMIs	1 (6.7)
Resistant to four drug classes, n (%)	1 (6.7)
Mecillinam + fluoroquinolones + cefpodoxime + FMIs	1 (6.7)

*DR1/2* resistant to one or two drug classes, *FMI* folate metabolism inhibitor, *MDR* resistant to three or more drug classes tested.

### AMR by region (secondary objective 1)

Overall AMR varied by region, as shown in Table 4. The Southeast had the highest proportion of patients with isolates classified as SUS (70.0%), the Northwest had the highest proportion of patients with isolates classified as DR1/2 (35.1%), and the Northeast had the highest proportion of patients with isolates classified as MDR (5.3%). The most notable differences in AMR across regions were observed for mecillinam (ranging from 8.4% in the Southeast to 28.6% in the Northeast) and FMIs (ranging from 12.7% in the Southwest to 26.1% in the Northeast).

**Table 4:** Antimicrobial resistance among non-pregnant female patients with uUTI who had information for ≥ 4 drug classes, stratified by regions in Germany.

Index drug class	All patients (N = 386)	Region				
		Northeast (n = 38)	Northwest (n = 57)	Southeast (n = 90)	Southwest (n = 119)	West (n = 82)
Fosfomycin						
Tested	373 (96.6)	37 (97.4)	56 (98.2)	88 (97.8)	113 (95.0)	79 (96.3)
Susceptible^a^	353 (94.6)	36 (97.3)	54 (96.4)	85 (96.6)	105 (92.9)	73 (92.4)
Resistant	20 (5.4)	1 (2.7)	2 (3.6)	3 (3.4)	8 (7.1)	6 (7.6)
Nitrofurantoin						
Tested	368 (95.3)	37 (97.4)	57 (100)	82 (91.1)	112 (94.1)	80 (97.6)
Susceptible^a^	344 (93.5)	37 (100)	56 (98.2)	77 (93.9)	101 (90.2)	73 (91.3)
Resistant	24 (6.5)	0 (0.0)	1 (1.8)	5 (6.1)	11 (9.8)	7 (8.8)
Mecillinam						
Tested	364 (94.3)	35 (92.1)	57 (100)	83 (92.2)	117 (98.3)	72 (87.8)
Susceptible^a^	319 (87.6)	25 (71.4)	52 (91.2)	76 (91.6)	102 (87.2)	64 (88.9)
Resistant	45 (12.4)	10 (28.6)	5 (8.8)	7 (8.4)	15 (12.8)	8 (11.1)
Fluoroquinolones^b^						
Tested	345 (89.4)	36 (94.7)	50 (87.7)	79 (97.8)	110 (92.4)	70 (85.4)
Susceptible^a^	327 (94.8)	31 (86.1)	45 (90.0)	74 (93.7)	107 (97.3)	70 (100)
Resistant^c^	18 (5.2)	5 (13.9)	5 (10.0)	5 (6.3)	3 (2.7)	0 (0.0)
Cefpodoxime						
Tested	347 (89.9)	34 (89.5)	50 (87.7)	84 (93.3)	106 (89.1)	73 (89.0)
Susceptible^a^	314 (90.5)	30 (88.2)	46 (92.0)	78 (92.9)	93 (87.7)	67 (91.8)
Resistant	33 (9.5)	4 (11.8)	4 (8.0)	6 (7.1)	13 (12.3)	6 (8.2)
FMIs^d^						
Tested	268 (69.4)	23 (60.5)	46 (80.7)	71 (78.9)	71 (59.7)	57 (69.5)
Susceptible^a^	219 (81.7)	17 (73.9)	37 (80.4)	56 (78.9)	62 (87.3)	47 (82.5)
Resistant^c^	49 (18.3)	6 (26.1)	9 (19.6)	15 (21.1)	9 (12.7)	10 (17.5)

All values shown are the n (%) of tested patients for whom the uropathogen (*E. coli*) was susceptible or resistant to the indicated drug class.

*E. coli* *Escherichia coli*, *FMI* folate metabolism inhibitor, *SXT* trimethoprim-sulfamethoxazole, *TMP* trimethoprim, *uUTI* uncomplicated urinary tract infection.

^a^Susceptible + intermediate/susceptible at higher dose.

^b^Included ciprofloxacin, levofloxacin, and ofloxacin.

^c^Uropathogens from patients were considered resistant to a drug class if they were resistant to ≥ 1 drug within the class.

^d^Included TMP and SXT.

### Treatment patterns (secondary objective 3)

Treatment dose and duration for antimicrobials used at index uUTI treatment were largely aligned with German UTI guidelines^15^ (Table 5). Overall, 385/386 (99.7%) patients were prescribed treatment within 28 days of the index date. Most patients (91.2%) received a single antimicrobial treatment; however, 8.8% (n = 34) received more than one antimicrobial class. More patients in the MDR cohort received a further treatment compared to the SUS cohort (26.7% vs 7.7%, respectively) (Table 5). Fluroquinolones (40.0%) and cefpodoxime (26.7%) were the most prescribed drugs/classes in the MDR cohort and were more likely to be prescribed (std. diffs = 54.9% and 41.5%, respectively) versus the SUS cohort, on or within 28 days of the index date (Supplementary Table 2). Fosfomycin was the most prescribed drug in the SUS (44.0%) and DR1/2 (41.4%) cohorts, but not in the MDR cohort (26.7%).

**Table 5:** Treatment patterns among non-pregnant female patients with uUTI and AMR information for ≥ 4 drug classes.

	All patients (N = 386)	Antimicrobial susceptibility cohort		
		SUS (n = 259)	DR1/2^a^ (n = 112)	MDR^a^ (n = 15)
Median (IQR) treatment courses prescribed on or within 28 days of the index date, n	1.0 (1.0, 1.0)	1.0 (1.0, 1.0)	1.0 (1.0, 1.0)	1.0 (1.0, 2.0)
Patients with drugs prescribed on or within 28 days of the index date, n (%)	385 (99.7)	259 (100)	111 (99.1)	15 (100)
Index drug class^b^				
***Fosfomycin-trometamol***, n (%)^c^	162 (42.1)	113 (43.6)	45 (40.5)	4 (26.7)
Median (IQR) duration of treatment, days	1.0 (1.0, 1.0)	1.0 (1.0, 1.0)	1.0 (1.0, 1.0)	1.0 (1.0, 1.0)
Median (IQR) daily dose, mg	3000 (3000, 3000)	3000 (3000, 3000)	3000 (3000, 3000)	3000 (3000, 3000)
***Nitrofurantoin***, n (%)^c^	64 (16.6)	44 (17.0)	17 (15.3)	3 (20.0)
Median (IQR) duration of treatment, days	5.0 (3.0, 7.0)	5.0 (3.0, 7.0)	5.0 (5.0, 7.0)	3.0 (3.0, 7.0)
Median (IQR) daily dose, mg	200 (200, 300)	200 (200, 300)	200 (125, 250)	300 (300, 300)
***Mecillinam***, n (%)^c^	31 (8.1)	19 (7.3)	11 (9.9)	1 (6.7)
Median (IQR) duration of treatment, days	3.0 (3.0, 5.0)	3.0 (3.0, 5.0)	3.0 (3.0, 3.0)	5.0 (5.0, 5.0)
Median (IQR) daily dose, mg	1200 (1200, 1500)	1200 (1200, 2000)	1200 (1200, 1200)	–
***Fluoroquinolones***, n (%)^c^	68 (17.7)	41 (15.8)	22 (19.8)	5 (33.3)
**Ciprofloxacin**, n (%)^c^	56 (14.5)	33 (12.7)	20 (18.0)	3 (20.0)
Median (IQR) duration of treatment, days	5.0 (4.0, 5.0)	5.0 (3.0, 5.0)	5.0 (5.0, 5.0)	5.0 (5.0, 5.0)
Median (IQR) daily dose, mg	500 (400, 1000)	550 (400, 1000)	500 (500, 750)	400 (400, 450)
**Levofloxacin**, n (%)^c^	11 (2.9)	9 (3.5)	1 (0.9)	1 (6.7)
Median (IQR) duration of treatment, days	5.0 (3.0, 5.0)	5.0 (3.0, 5.0)	1.0 (1.0, 1.0)	7.0 (7.0, 7.0)
Median (IQR) daily dose, mg	500 (500, 500)	500 (500, 500)	–	500 (500, 500)
**Ofloxacin**, n (%)^c^	2 (0.5)	0	1 (0.9)	1 (6.7)
Median (IQR) duration of treatment, days	6.0 (5.0, 7.0)	–	5.0 (5.0, 5.0)	7.0 (7.0, 7.0)
Median (IQR) daily dose, mg	400 (400, 400)	–	–	400 (400, 400)
***Cefpodoxime***, n (%)^c^	40 (10.4)	27 (10.4)	11 (9.9)	2 (13.3)
Median (IQR) duration of treatment, days	5.0 (5.0, 6.5)	5.0 (5.0, 5.0)	5.0 (5.0, 7.0)	4.0 (3.0, 5.0)
Median (IQR) daily dose, mg	750 (400, 1000)	1000 (500, 1000)	500 (400, 1000)	400 (400, 400)
***FMIs***, n (%)^c^	27 (7.0)	19 (7.3)	7 (6.3)	1 (6.7)
**TMP**, n (%)^c^	6 (1.6)	6 (2.3)	0	0
Median (IQR) duration of treatment, days	5.0 (3.0, 5.0)	5.0 (3.0, 5.0)	–	–
Median (IQR) daily dose, mg	350 (200, 400)	350 (200, 400)	–	–
**SMX**, n (%)^c^	21 (5.5)	13 (5.0)	7 (6.3)	1 (6.7)
Median (IQR) duration of treatment, days	5.0 (5.0, 5.0)	5.0 (5.0, 5.0)	5.0 (5.0, 5.0)	7.0 (7.0, 7.0)
SMX 160/800 mg BID, n (%)^d^	15 (93.8) (n = 16)	10 (90.9) (n = 11)	4 (100) (n = 4)	1 (100) (n = 1)

Per the 2017 Update of the German Clinical Guideline on Epidemiology, Diagnostics, Therapy, Prevention, and Management of uUTI in Adult Patients (Part II: Therapy and Prevention), the recommended empirical short-term antimicrobial treatment duration for the tested drug classes is 1 day for fosfomycin-trometamol; 3 days for TMP, mecillinam, cefpodoxime, and fluoroquinolones; and 7 days for nitrofurantoin^15^.

*AMR* antimicrobial resistance, *BID* twice daily, *DR1/2* resistant to one or two drug classes, *FMI* folate metabolism inhibitor, *IQR* interquartile range, *MDR* resistant to three or more drug classes tested, *SUS* susceptible, *SMX* sulfamethoxazole, *TMP* trimethoprim, *uUTI* uncomplicated urinary tract infection.

^a^Uropathogens from patients were considered resistant to a drug class if they were resistant to ≥ 1 drug within the class.

^b^Drug names are shown in bold, drug classes are shown in bold italics.

^c^Calculated as % of patients with antimicrobial treatment prescribed on or within 28 days of the index date (numbers restricted to index antimicrobial treatment only).

^d^Calculated as % of patients with evaluable dosage information (n) for SMX 160/800 mg BID.

### Clinical outcomes (secondary objective 4)

Treatment failure and uUTI recurrence (≥ 2 uUTIs in 6-months or ≥ 3 uUTIs in 1 year during the baseline period) varied by AMR status. Initial antimicrobial treatment for uUTI failed for 34/386 (8.8%) patients (Table 6). The proportion of patients experiencing treatment failure in each cohort was: SUS 7.7%, DR1/2 8.9%, and MDR 26.7%. In adjusted comparative analyses, treatment failure was higher in the MDR versus SUS cohort [OR (95% CI) = 4.21 (1.14–15.50); P = 0.031]. Overall, the proportion of patients with treatment failure was higher among patients with a history of recurrent uUTI than in patients who had non-recurrent uUTI at index (15.9% and 7.4%, respectively). The incidence rate of recurrent uUTI within 6-months post index was significantly higher in the DR1/2 versus SUS cohort [IRR (95% CI) = 1.67 (1.00–2.77); P = 0.046]. The difference in incidence rate of recurrent infections between the MDR and SUS cohorts was not statistically significant. Furthermore, no significant differences between cohorts were seen in the incidence rate of recurrent infections within 12-months of index, or in the odds of experiencing recurrent infections within 6 or 12-months of index. Overall, 43 (11.1%) patients had at least one complication within 28 days of the index date. The most frequent complications observed were fever (n = 30; 7.8%) and progression to complicated UTI (n = 22; 5.7%). No significant differences in the number of complications were observed between the SUS and DR1/2 cohorts. No patients in the MDR cohort experienced complications within 28 days of index; however, the sample size was very small (Table 6). During the observation period, there were six patients (10.5%) with all-cause hospitalization and one of these patients had a UTI-related hospitalization. However, it was not established if patients were hospitalized because of UTI complications or adverse effects of UTI treatment. The average length of stay was 6.2 days for the six patients.

**Table 6:** Clinical outcomes among non-pregnant female patients with uUTI and AMR information for ≥ 4 drug classes.

	Descriptive statistics				Multivariable analysis^a^			
	All patients (N = 386)	[1] SUS (n = 259)	[2] DR1/2^b^ (n = 112)	[3] MDR^b^ (n = 15)				
					[2] vs [1] Adjusted OR (95% CI)	P value	[3] vs [1] Adjusted OR (95% CI)	P value
Binary outcomes^c^								
Treatment failure^d^, n (%)	34 (8.8)	20 (7.7)	10 (8.9)	4 (26.7)	1.34 (0.60–3.04)	0.476	4.21 (1.14–15.50)	0.031^e^
Recurrent infections^f^, n (%)								
Within 6-months of index date	52 (13.5)	28 (10.8)	20 (17.9)	4 (26.7)	1.92 (0.97–3.79)	0.060	2.23 (0.62–8.06)	0.220
Within 12-months of index date	110 (28.5)	69 (26.6)	35 (31.3)	6 (40.0)	1.38 (0.79–2.41)	0.253	1.16 (0.35–3.86)	0.806
Complications within 28 days of index, n (%)								
Any	43 (11.1)	28 (10.8)	15 (13.4)	Unobserved	1.30 (0.64–2.63)	0.469	NA	NA
Fever	30 (7.8)	22 (8.5)	8 (7.1)	Unobserved	0.83 (0.35–1.97)	0.681	NA	NA
Complicated UTI	22 (5.7)	15 (5.8)	7 (6.3)	Unobserved	1.07 (0.39–2.88)	0.899	NA	NA
Acute pyelonephritis	4 (1.0)	1 (0.4)	3 (2.7)	Unobserved	8.61 (0.85–86.83)	0.068	NA	NA
Sepsis	1 (0.3)	1 (0.4)	Unobserved	Unobserved	NA	NA	NA	NA

Count outcomes^g^ IR of recurrent infections^f,h^, per person-year					Adjusted IRR (95% CI)	P value	Adjusted IRR (95% CI)	P value
Within 6-months of index date	0.35	0.28	0.46	0.67	1.67 (1.00–2.77)	0.048^e^	1.86 (0.71–4.86)	0.205
Within 12-months of index date	0.44	0.39	0.51	0.60	1.31 (0.95–1.81)	0.104	1.13 (0.56–2.26)	0.730

Standardized difference > 20%, > 50%, and > 80% denotes a small, medium, and large difference, respectively, between compared groups (per Cohen J. Statistical Power Analysis for the Behavioral Sciences. 2nd ed.; 1988).

*AMR* antimicrobial resistance, *CI* confidence interval, *DR1/2* resistant to one or two drugs, *IR* incidence rate, *IRR* incidence rate ratio, *MDR* resistant to three or more drug classes tested, *NA* not applicable, *OR* odds ratio, *SUS* susceptible, *UTI* urinary tract infection, *uUTI* uncomplicated urinary tract infection.

^a^Adjusted for age, prior history of UTI, and prior history of antimicrobial treatment during the baseline period (i.e., 12-month period prior to the index date), with SUS as the reference cohort.

^b^Uropathogens from patients were considered resistant to a drug class if they were resistant to ≥ 1 drug within the class.

^c^Logistic regressions with robust variance estimator used to derive OR estimates and associated CIs.

^d^Treatment failure was defined as a subsequent prescription of an antimicrobial within 28 days of the index date.

^e^P value < 0.05.

^f^Recurrent infection was defined as a new diagnosis of uUTI occurring after 28 days following the index date.

^g^Poisson regressions with robust variance estimator used to derive IRR estimates and associated CIs.

^h^The IR was calculated as the frequency of recurrent infections divided by the total person-years of observation.

## Discussion

Optimizing treatment for uUTI requires up-to-date surveillance of overall and regional AMR, as well as real-world data on treatment patterns and clinical outcomes. However, such data were outdated or not readily available in Germany. In this context, this study generated detailed real-world data on overall and regional prevalence of AMR, patient characteristics by AMR status, treatment patterns, and clinical outcomes. Women were exclusively included in this study as the indication of interest was AUC, which only occurs in females per both US and EU treatment guidelines^4,20^. Acute cystitis in males is considered to be complicated UTI^4,20^. Adolescents aged ≥ 12 years were eligible because the epidemiology of AUC is similar for adolescent and adult females. *E. coli* was evaluated exclusively because it is the most important uropathogen; *E. coli* is responsible for 70–90% of AUC isolates^21,22^ and a significant proportion of AMR in the community setting.

The study had four main findings. First, AMR increased during the study period and varied by drug class and across regions. Second, there were age, comorbidity, and baseline clinical differences by AMR status. Third, treatment patterns largely followed German UTI treatment guidelines^5^, with some exceptions for fluoroquinolones and cefpodoxime-proxetil. Finally, treatment failure and UTI recurrence varied by AMR status.

Compared to a study of AMR in patients with uUTI in Germany by Seitz et al., conducted from 2015–2017^11^, in our study, the proportions of patients with resistance to cefpodoxime, fosfomycin, and nitrofurantoin were higher, resistance to FMIs was similar, and resistance to ciprofloxacin was lower. While an increase of AMR over time is not unexpected, comparisons of our findings with prior studies need to be interpreted with caution. First, data sources were different. While our study included data from multiple clinics across medical specialties and regions in Germany, Seitz et al. only included data from one urological practice in Munich, and therefore these results may not be as generalizable as our findings across Germany. Second, a major change happened to the EUCAST guidelines in 2019^14^, which provides important context regarding the interpretation of susceptibility and resistance over time: isolates that were “intermediate” or “susceptible at higher dose” were considered “not-susceptible” prior to the change but considered “susceptible” after this change. Consequently, resistance rates found in our study, using the 2019 EUCAST guidelines^14^, are conservatively lower than they would be had the EUCAST definition remained constant. Finally, the lower resistance rate found for ciprofloxacin in our study compared to Seitz et al. may be due to EU restrictions in the use of fluoroquinolones in 2019 after safety warnings due to the risk of serious adverse events^23^. Changes to antimicrobial EUCAST breakpoints between 2017 and 2019 may have affected the results of this study. In 2019, the ciprofloxacin zone diameter breakpoint was reduced, resulting in an increased number of patients categorized as susceptible^14,24,25^. Nonetheless, the present study finds that the percentage of patients with susceptible pathogens declined between 2017 and 2019. The EUCAST UTI breakpoint for fosfomycin resistance was lowered in 2021^26^. Therefore, if defined using the new breakpoint, the fosfomycin resistance rates reported in our study would likely be higher.

Several studies showing differences in AMR trends between Germany and other countries have been conducted^3,16,17,19,27^, but there was little evidence of regional variation in AMR within Germany. Our study found that overall AMR varied by German region: these findings complemented prior research showing differences in uUTI prescribing practices by German region in 2008^16^. However, in contrast with this study, our study found no major difference in prescribing patterns by physician specialty (general practitioner and specialist)^16^. Differences in demographic and patient characteristics across antimicrobial susceptibility cohorts were observed, consistent with a prior study that found associations between antimicrobial susceptibility and patient characteristics such as age and sex^28^. In contrast with prior studies highlighting low compliance with prescribing guidelines^16,29^, our study found that treatment dosing and duration for each drug class largely aligned with German UTI treatment guidelines. In our study, treatment failure was more likely in the MDR versus SUS cohorts, and incidence of recurrent infection in the 6-months post index was higher in the DR1/2 versus SUS cohorts. Prior research also found that patients with recurrent UTIs had different susceptibility profiles compared with those of patients with non-recurrent infections^30^. Further investigation of the relationship between recurrent uUTIs and AMR is warranted.

The strength of this study lies in the generation of granular, real-world data on uUTI treatment patterns, and clinical outcomes from physicians with direct insight into patient medical history and the clinical course of uUTIs. Although less resource-intensive than a prospective cohort study, the study design allowed for the collection of up-to-date, clinically relevant information, not readily available in existing databases. Similar studies in other countries would help better understand the global burden of AMR.

This study also has limitations. As with all chart review studies, measurement error in data collection cannot be ruled out. Statistical inference based on retrospectively collected data may have been affected by potential confounding and selection bias. However, AMR prevalence estimation was not influenced by the exclusion of patients with negative urine cultures, as these patients (negative cultures) do not contribute to AMR assessment. The study was also not powered to detect differences between the cohorts for secondary endpoints.

The study results are generalizable to female patients in Germany with AUC who have a positive urine culture caused by *E. coli*. However, the study results may not be generalizable to all females with AUC in Germany, including those who did not receive urine cultures or those caused by other uropathogens.

The requirement of a 12-month baseline period had the potential to introduce selection bias into the study. However, the lack of this baseline period could have led to misclassification of AUC. The inclusion of a baseline period was deemed more important given that AUC misclassification leading to the inclusion of complicated UTI would have overestimated AMR prevalence and undermined external validity. Furthermore, a 12-month baseline period is a common requirement in observational studies and is used to assess underlying comorbidities and apply exclusion criteria for urological abnormalities associated with complicated UTI^31,32^. The clinically relevant time-period for assessing outcomes of the index case was 28 days post-index. There were no other noted infections ongoing at the time of re-prescription and re-prescriptions were recorded as prescriptions for uUTI infections per electronic case report form.

The minimum inhibitory concentrations used in the antimicrobial susceptibility tests were not available in most patient charts and were not collected. In addition, there was no information on how susceptibility testing was performed or reported at the laboratory level and there was potential for variation across laboratories in different regions (the exact method used is not recorded in patient charts; however, the methods are standardized and follow EUCAST guidelines). This may have led to bias if different regions had different approaches to susceptibility testing. We are not aware of cascade reporting in Germany, but AMR may have been underestimated if cascade reporting suppressed the results for a specific drug class.

## Conclusions

Our study showed that among patients with uUTI in Germany, one-third of *E. coli* urine isolates were resistant to at least one of the tested antimicrobial drug classes. However, it is worth noting that overall antibiotic resistance for fosfomycin and nitrofurantoin remained low. AMR increased over time and varied by drug class and across regions. These findings highlight the need for ongoing, regular community monitoring of uUTIs in Germany, and a need for novel antimicrobials to help overcome antimicrobial resistant *E. coli* in uUTI. Future research aiming to inform antimicrobial prescribing should explore AMR in uUTI in other countries and investigate the relationship between recurrent UTIs and AMR.

## Supplementary Information

Supplementary Tables.

## Data Availability

The datasets generated and/or analysed during the current study are not publicly available due to patient data privacy per General Data Protection Regulation in the EU, but are available from the corresponding author on reasonable request.

## References

[1] Foxman B (2002). Epidemiology of urinary tract infections: Incidence, morbidity, and economic costs. Am. J. Med..

[2] Medina M, Castillo-Pino E (2019). An introduction to the epidemiology and burden of urinary tract infections. Ther. Adv. Urol..

[3] Wagenlehner F (2021). A global perspective on improving patient care in uncomplicated urinary tract infection: Expert consensus and practical guidance. J. Glob. Antimicrob. Resist..

[4] Bonkat, G. *et al. EAU Guidelines on Urological Infections*. https://d56bochluxqnz.cloudfront.net/documents/full-guideline/EAU-Guidelines-on-Urological-infections-2023.pdf (2023).

[5] Kranz J (2018). The 2017 update of the German clinical guideline on epidemiology, diagnostics, therapy, prevention, and management of uncomplicated urinary tract infections in adult patients: part 1. Urol. Int..

[6] Wagenlehner FM (2011). Uncomplicated urinary tract infections. Dtsch. Arztebl. Int..

[7] Ventola CL (2015). The antibiotic resistance crisis: Part 1: Causes and threats. Pharmacol. Ther..

[8] Prestinaci F, Pezzotti P, Pantosti A (2015). Antimicrobial resistance: A global multifaceted phenomenon. Pathog. Glob. Health.

[9] Federal Office of Consumer Protection and Food Safety (Paul-Ehrlich-Gesellschaft für Chemotherapie e.V.). *GERMAP 2015—Report on the Consumption of Antimicrobials and the Spread of Antimicrobial Resistance in Human and Veterinary Medicine in Germany. Antiinfectives Intelligence, Rheinbach, 2016*. https://www.bvl.bund.de/SharedDocs/Downloads/05_Tierarzneimittel/germap2015_EN.pdf?__blob=publicationFile&v=52016 (2016).

[10] Schmiemann G, Gagyor I, Hummers-Pradier E, Bleidorn J (2012). Resistance profiles of urinary tract infections in general practice—An observational study. BMC Urol..

[11] Seitz M, Stief C, Waidelich R (2017). Local epidemiology and resistance profiles in acute uncomplicated cystitis (AUC) in women: A prospective cohort study in an urban urological ambulatory setting. BMC Infect. Dis..

[12] Murray CJ (2022). Global burden of bacterial antimicrobial resistance in 2019: A systematic analysis. Lancet.

[13] OECD. *Stemming the Superbug Tide*. https://www.oecd.org/els/health-systems/Stemming-the-Superbug-Tide-Policy-Brief-2018.pdf (2018).

[14] The European Committee on Antimicrobial Susceptibility Testing. *Breakpoint Tables for Interpretation of MICs and Zone Diameters. Version 9.0, 2019.* https://www.eucast.org/fileadmin/src/media/PDFs/EUCAST_files/Breakpoint_tables/v_9.0_Breakpoint_Tables.pdf (2019).

[15] Kranz J (2018). The 2017 update of the German clinical guideline on epidemiology, diagnostics, therapy, prevention, and management of uncomplicated urinary tract infections in adult patients: Part II: Therapy and prevention. Urol. Int..

[16] Naber KG, Schito G, Botto H, Palou J, Mazzei T (2008). Surveillance study in Europe and Brazil on clinical aspects and Antimicrobial Resistance Epidemiology in Females with Cystitis (ARESC): Implications for empiric therapy. Eur. Urol..

[17] Schito GC (2009). The ARESC study: An international survey on the antimicrobial resistance of pathogens involved in uncomplicated urinary tract infections. Int. J. Antimicrob. Agents.

[18] Kahlmeter G, Menday P (2003). Cross-resistance and associated resistance in 2478 *Escherichia coli* isolates from the Pan-European ECO· SENS Project surveying the antimicrobial susceptibility of pathogens from uncomplicated urinary tract infections. J. Antimicrob. Chemother..

[19] Wagenlehner FM (2010). Clinical aspects and epidemiology of uncomplicated cystitis in women. German results of the ARESC Study. Urologe A.

[20] Gupta K (2011). International clinical practice guidelines for the treatment of acute uncomplicated cystitis and pyelonephritis in women: A 2010 update by the Infectious Diseases Society of America and the European Society for Microbiology and Infectious Diseases. Clin. Infect. Dis..

[21] Lee DS, Lee SJ, Choe HS (2018). Community-acquired urinary tract infection by *Escherichia coli* in the era of antibiotic resistance. Biomed. Res. Int..

[22] Foroughi A, Ramezan-Ghanbari S (2020). The frequency of Stx1 and Stx2 genes in uropathogenic *Escherichia coli i*solated from patients in Kermanshah, Iran. Epigenetics.

[23] EMA. *Disabling and Potentially Permanent Side Effects Lead to Suspension or Restrictions of Quinolone and Fluoroquinolone Antibiotics*. https://www.ema.europa.eu/en/documents/referral/quinolone-fluoroquinolone-article-31-referral-disabling-potentially-permanent-side-effects-lead_en.pdf (2019).

[24] The European Committee on Antimicrobial Susceptibility Testing. *Breakpoint Tables for Interpretation of MICs and Zone Diameters. Version 7.1, 2017*. https://www.eucast.org/fileadmin/src/media/PDFs/EUCAST_files/Breakpoint_tables/v_7.1_Breakpoint_Tables.pdf (2017).

[25] The European Committee on Antimicrobial Susceptibility Testing. *Breakpoint Tables for Interpretation of MICs and Zone Diameters. Version 8.1, 2018*. https://www.eucast.org/fileadmin/src/media/PDFs/EUCAST_files/Consultation/2018/Information_for_NACs_-_Redefining_antimicrobial_susceptibility_testing_categories.pdf (2018).

[26] The European Committee on Antimicrobial Susceptibility Testing. *Breakpoint Tables for Interpretation of MICs and Zone Diameters. Version 11.0, 2021*. https://www.eucast.org/fileadmin/src/media/PDFs/EUCAST_files/Breakpoint_tables/v_11.0_Breakpoint_Tables.pdf (2021).

[27] Kahlmeter G, Poulsen HO (2012). Antimicrobial susceptibility of *Escherichia coli* from community-acquired urinary tract infections in Europe: The ECO.SENS study revisited. Int. J. Antimicrob. Agents.

[28] Erb S (2018). Basic patient characteristics predict antimicrobial resistance in *E. coli* from urinary tract specimens: A retrospective cohort analysis of 5246 urine samples. Swiss Med. Wkly..

[29] Zweigner J, Meyer E, Gastmeier P, Schwab F (2018). Rate of antibiotic prescriptions in German outpatient care—Are the guidelines followed or are they still exceeded?. GMS Hyg. Infect. Control.

[30] Hisano M (2015). The bacterial spectrum and antimicrobial susceptibility in female recurrent urinary tract infection: How different they are from sporadic single episodes?. Urol. J..

[31] Aypak C, Altunsoy A, Duzgun N (2009). Empiric antibiotic therapy in acute uncomplicated urinary tract infections and fluoroquinolone resistance: A prospective observational study. Ann. Clin. Microbiol. Antimicrob..

[32] Daneman N (2020). Fluoroquinolone use for uncomplicated urinary tract infections in women: A retrospective cohort study. Clin. Microbiol. Infect..

