# Supplementary material for: Escherichia coli resistance, treatment patterns and clinical outcomes among females with uUTI in Germany: a retrospective physician-based chart review study
Source: Sci Rep. 2023 Jul 26;13:12077. doi: 10.1038/s41598-023-38919-8 (PMC10372039; doi:10.1038/s41598-023-38919-8)
Supplement: Supplementary file 1 — Supplementary Tables. [file 41598_2023_38919_MOESM1_ESM.pdf]

# Uncomplicated urinary tract infections among female patients in Germany: a retrospective physician-based chart review study

Kurt G. Naber[1] · Florian Wagenlehner[2] · Michael Kresken[3] · Wendy Y. Cheng[4] · Maryaline Catillon[4] · Mei Sheng Duh[4] · Louise Yu[4] · Anamika Khanal[4] · Aruni Mulgirigama[5] · Ashish V. Joshi[6] · Shinyoung Ju[5] · Fanny S. Mitrani-Gold[6]

[1] Technical University of Munich, Munich, Germany

[2] Clinic of Urology, Pediatric Urology and Andrology, Justus Liebig University Giessen, Germany

[3] Antiinfectives Intelligence GmbH, Cologne, Germany

[4] Analysis Group, Inc., Boston, MA, USA

[5] GSK, Brentford, Middlesex, UK

[6] GSK, Collegeville, PA, USA

**Corresponding author:** Fanny S. Mitrani-Gold, GSK, 1250 S Collegeville Road, Collegeville, PA 19426, USA; fanny.s.mitrani-gold@gsk.com

## SUPPLEMENTARY MATERIALS

**Supplementary Table 1** Histories of antimicrobial treatments, allergies and resistance among non-pregnant patients with uUTI and AMR information for ≥ 4 drug classes

| | All patients (*N* = 386) | [1] SUS (*n* = 259) | [2] DR1/2[a] (*n* = 112) | [3] MDR[a] (*n* = 15) | *Std. diff, %*[b, c] | |
|---|---|---|---|---|---|---|
| | | | | | [1] vs. [2] | [1] vs. [3] |
| Prior history of antimicrobial treatments during 12-month baseline period by drug class[d] | | | | | | |
| *Fosfomycin trometamol, n (%)* | 65 (16.8) | 43 (16.6) | 20 (17.9) | 2 (13.3) | 3.3 | 9.2 |
| *Nitrofurantoin, n (%)* | 27 (7.0) | 16 (6.2) | 11 (9.8) | 0 (0.0) | 13.5 | 36.3[e] |
| *Extended spectrum penicillins, n (%)* | 10 (2.6) | 5 (1.9) | 4 (3.6) | 1 (6.7) | 10.1 | 23.5[e] |
| Pivmecillinam | 9 (2.3) | 4 (1.5) | 4 (3.6) | 1 (6.7) | 12.9 | 26.0[e] |
| *Fluoroquinolones*, n (%) | 21 (5.4) | 16 (6.2) | 4 (3.6) | 1 (6.7) | 12.1 | 2.0 |
| Ciprofloxacin | 19 (4.9) | 14 (5.4) | 4 (3.6) | 1 (6.7) | 8.9 | 5.3 |
| Levofloxacin | 3 (0.8) | 2 (0.8) | 1 (0.9) | 0 (0.0) | 1.3 | 12.5 |
| Ofloxacin | 0 (0.0) | 0 (0.0) | 0 (0.0) | 0 (0.0) | - | - |
| *Third generation cephalosporins*, n (%) | 10 (2.6) | 5 (1.9) | 4 (3.6) | 1 (6.7) | 10.1 | 23.5[e] |
| Cefpodoxime | 10 (2.6) | 5 (1.9) | 4 (3.6) | 1 (6.7) | 10.1 | 23.5[e] |
| *FMIs,* n (%) | 6 (1.6) | 4 (1.5) | 2 (1.8) | 0 (0.0) | 1.9 | 17.7 |
| TMP | 1 (0.3) | 1 (0.4) | 0 (0.0) | 0 (0.0) | 8.8 | 8.8 |
| SXT | 5 (1.3) | 3 (1.2) | 2 (1.8) | 0 (0.0) | 5.2 | 15.3 |
| Other antimicrobial treatments[f], n (%) | 4 (1.0) | 3 (1.2) | 1 (0.9) | 0 (0.0) | 2.6 | 15.3 |
| Other[g] | 1 (0.3) | 1 (0.4) | 0 (0.0) | 0 (0.0) | 8.8 | 8.8 |
| Any antimicrobial treatment prescribed within 6 months prior to the index date[h], n (%) | 57 (14.8) | 40 (15.4) | 15 (13.4) | 2 (13.3) | 5.8 | 6.0 |
| Prior history of antimicrobial allergy/intolerance at index, n (%) | | | | | | |
| Fosfomycin | 17 (4.4) | 11 (4.2) | 6 (5.4) | 0 (0.0) | 5.2 | 29.8[e] |
| Nitrofurantoin | 8 (2.1) | 6 (2.3) | 2 (1.8) | 0 (0.0) | 3.8 | 21.8[e] |
| Pivmecillinam | 14 (3.6) | 9 (3.5) | 4 (3.6) | 1 (6.7) | 0.5 | 14.6 |
| *Fluoroquinolones* | 4 (1.0) | 2 (0.8) | 2 (1.8) | 0 (0.0) | 9.0 | 12.5 |
| Cefpodoxime | 5 (1.3) | 4 (1.5) | 1 (0.9) | 0 (0.0) | 5.9 | 17.7 |
| FMIs | 2 (0.5) | 1 (0.4) | 1 (0.9) | 0 (0.0) | 6.4 | - |
| Prior history of antimicrobial resistance at index, n (%) | | | | | | |
| UTI-specific antimicrobials | 7 (1.8) | 5 (1.9) | 1 (0.9) | 1 (6.7) | 8.8 | 23.5[e] |
| Fosfomycin | 5 (1.3) | 3 (1.2) | 1 (0.9) | 1 (6.7) | 2.6 | 28.7[e] |
| Nitrofurantoin | 5 (1.3) | 4 (1.5) | 1 (0.9) | 0 (0.0) | 5.9 | 17.7 |

| | | | | | | |
|---|---|---|---|---|---|---|
| Quinolones | 15 (3.9) | 5 (1.9) | 9 (8.0) | 1 (6.7) | 28.3[e] | 23.5[e] |
| Cephalosporins and other beta-lactams | 7 (1.8) | 3 (1.2) | 3 (2.7) | 1 (6.7) | 11.1 | 28.7[e] |
| Penicillins | 27 (7.0) | 13 (5.0) | 11 (9.8) | 3 (20.0) | 18.4 | 46.5[e] |
| Tetracyclines | 6 (1.6) | 1 (0.4) | 4 (3.6) | 1 (6.7) | 23.0[e] | 34.6[e] |
| Macrolides | 5 (1.3) | 1 (0.4) | 4 (3.6) | 0 (0.0) | 23.0[e] | 8.8 |
| Sulfonamides and TMP | 17 (4.4) | 6 (2.3) | 9 (8.0) | 2 (13.3) | 26.0[e] | 41.9[e] |
| Metronidazole, tinidazole, and ornidazole | 3 (0.8) | 2 (0.8) | 1 (0.9) | 0 (0.0) | 1.3 | 12.5 |
| Clindamycin and lincomycin | 6 (1.6) | 2 (0.8) | 4 (3.6) | 0 (0.0) | 19.3 | 12.5 |

Standardized difference >20%, >50%, and >80% denotes a small, medium, and large difference, respectively, between compared groups (per Cohen J. Statistical Power Analysis for the Behavioral Sciences. 2nd ed.; 1988). [a]Uropathogens from patients were considered resistant to a drug class if they were resistant to ≥1 drug within the class. [b]For continuous variables, the standardized difference was calculated by dividing the absolute difference in means between cohorts by the pooled standard deviation of both groups (the pooled standard deviation was the square root of the average of the squared standard deviations). [c]For dichotomous variables, the standardized difference was calculated using the following equation where P was the respective proportion of participants in each group: |(Pcase-Pcontrol)| / √[(Pcase(1-Pcase)+Pcontrol(1-Pcontrol))/2]. [d]Patients might have more than 1 clinical characteristic documented or received more than 1 treatment. [e]Standardized difference >20%. [f]Included cefuroxime. [g]Included amoxicillin. [h]Assessed among patients with prescription date of treatment available. *AMR* antimicrobial resistance, *DR1/2* resistant to 1 or 2 drugs, *FMI* folate metabolism inhibitors, *MDR* resistant to 3 or more drugs tested, *std. diff* standardized difference, *SUS* susceptible, *SXT* trimethoprim sulfamethoxazole, *TMP* trimethoprim, *UTI* urinary tract infection, *uUTI* uncomplicated urinary tract infection

**Supplementary Table 2** Treatment patterns among non-pregnant females with uUTI and AMR information for ≥ 4 drug classes on or within 28 days of the index date

| | **All patients (*N* = 386)** | **[1] SUS (*n* = 259)** | **[2] DR1/2[a] (*n* = 112)** | **[3] MDR[a] (*n* = 15)** | ***Std. diff, %*[b,c]** | |
|---|---|---|---|---|---|---|
| | | | | | **[2] vs. [1]** | **[3] vs. [1]** |
| Mean (SD) number[d] of antimicrobial treatments prescribed on or within 28 days of the index date | 1.1 (0.4) | 1.1 (0.4) | 1.1 (0.4) | 1.4 (0.7) | 0.7 | 48.1[e] |
| Median (IQR) number[d] of antimicrobial treatments prescribed on or within 28 days of the index date | 1.0 (1.0, 1.0) | 1.0 (1.0, 1.0) | 1.0 (1.0, 1.0) | 1.0 (1.0, 2.0) | - | - |
| **Drug class prescribed on or within 28 days of the index date[f], n (%)** | 385 (99.7) | 259 (100.0) | 111 (99.1) | 15 (100.0) | 13.42 | 0.0 |
| Fosfomycin | 164 (42.6) | 114 (44.0) | 46 (41.4) | 4 (26.7) | 5.2 | 36.9[e] |
| Nitrofurantoin | 68 (17.7) | 46 (17.8) | 19 (17.1) | 3 (20.0) | 1.7 | 5.7 |
| Pivmecillinam | 33 (8.6) | 20 (7.7) | 11 (9.9) | 2 (13.3) | 7.7 | 18.4 |
| *Fluoroquinolones* | 70 (18.2) | 42 (16.2) | 22 (19.8) | 6 (40.0) | 9.4 | 54.9[g] |
| Ciprofloxacin | 58 (15.1) | 34 (13.1) | 20 (18.0) | 4 (26.7) | 13.5 | 34.4[e] |
| Levofloxacin | 12 (3.1) | 9 (3.5) | 2 (1.8) | 1 (6.7) | 10.5 | 14.6 |
| Ofloxacin | 2 (0.5) | 0 (0.0) | 1 (0.9) | 1 (6.7) | 13.5 | 37.8[e] |
| Cefpodoxime | 43 (11.2) | 28 (10.8) | 11 (9.9) | 4 (26.7) | 3.0 | 41.5[e] |
| *FMIs* | 30 (7.8) | 21 (8.1) | 8 (7.2) | 1 (6.7) | 3.4 | 5.5 |
| TMP | 8 (2.1) | 8 (3.1) | 0 (0.0) | 0 (0.0) | 25.3[e] | 25.3[e] |
| SXT | 22 (5.7) | 13 (5.0) | 8 (7.2) | 1 (6.7) | 9.1 | 7.0 |
| Other antimicrobial treatments[h] | 4 (1.0) | 4 (1.5) | 0 (0.0) | 0 (0.0) | 17.7 | 17.7 |

Standardized difference >20%, >50%, and >80% denotes a small, medium, and large difference, respectively, between compared groups (per Cohen J. Statistical Power Analysis for the Behavioral Sciences. 2nd ed.; 1988). [a]Uropathogens from patients were considered resistant to a drug class if they were resistant to ≥ 1 drug within the class. [b]For continuous variables, the standardized difference was calculated by dividing the absolute difference in means between cohorts by the pooled standard deviation of both groups (the pooled standard deviation was the square root of the average of the squared standard deviations). [c]For dichotomous variables, the standardized difference was calculated using the following equation where P was the respective proportion of participants in each group: $|(P_{case}-P_{control})| / \sqrt{[(P_{case}(1-P_{case})+P_{control}(1-P_{control}))/2]}$. [d]Calculated at the drug level, including retreatment with the same antimicrobial. [e]Standardized difference >20%. [f]Patients might be prescribed ≥ 1 type of antimicrobial treatment on or within 28 days of the index date. [g]Standardized difference >50%. [h]Included cefuroxime, ampicillin/sulbactam and amoxicillin/clavulanic acid. *AMR* antimicrobial resistance, *DR1/2* resistant to 1 or 2 drugs, *FMI* folate metabolism inhibitor, *IQR* interquartile range, *MDR* resistant to 3 or more drug classes tested, *SD* standard deviation, *std. diff* standardized difference, *SUS* susceptible, *SXT* trimethoprim sulfamethoxazole, *TMP* trimethoprim, *uUTI* uncomplicated urinary tract infection
